# Safety of high‐dose *Puerariae Lobatae Radix* in adolescent rats based on metabolomics

**DOI:** 10.1002/fsn3.2044

**Published:** 2020-12-01

**Authors:** Limei Chen, E. Jiang, Yongmei Guan, Pan Xu, Qian Shen, Zhiyong Liu, Weifeng Zhu, Lihua Chen, Hongning Liu, Huanhuan Dong

**Affiliations:** ^1^ Jiangxi University of Traditional Chinese Medicine Nanchang China

**Keywords:** adolescent rat, metabolomics, *Puerariae Lobatae Radix*, safety

## Abstract

*Puerariae Lobatae Radix (PLR)* is the dried root of the leguminous plant *Pueraria lobata* and is a common component of health products and medicines. Although it is considered safe, some studies have reported that *PLR* has hepatotoxicity and estrogen‐like effects. In this study, the safety of high doses of *PLR* water extract administered to adolescent *SD* rats for 30 days was evaluated by biochemical, histopathological, and metabolomic analyses. Overall, there were no significant differences between the low‐dose and blank control groups in parameter values, including organ wet weight, organ coefficient, routine blood indicators, serum biochemical indexes of liver and renal function, levels of estradiol and testosterone, histopathological parameters, and primary differential metabolite profiles. Compared with the blank control group, the high‐dose group may have a certain effect on the liver. These effects might be mediated by abnormal phenylalanine, tyrosine, and tryptophan biosynthesis or phenylalanine metabolism. However, histopathological analyses did not show differences in the liver, kidney, breast, uterus, ovary, testis, and epididymis between the control group and the group treated with a high dose of *PLR* water extract. *PLR* water extract did not significantly promote the precocity of male and female sexual organs. Overall, *PLR* water extract is relatively safe for adolescent *SD* rats.

## Graphical abstract

*Pueraria lobata* can be used as a medicine and as a food. It has high safety and can be used for daily consumption. It can be used in the development of health food and medicine, but it contains more phytoestrogens (isoflavones) which may have a certain impact on the adolescent population. We evaluated the safety of large‐dose Chinese medicine *Pueraria lobata* for puberty by feeding adolescent male and female *SD* rats. This research found that *Puerariae Lobatae Radix* water extract was relatively safe for adolescent *SD* rats.

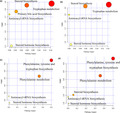

## 1 INTRODUCTION

*Puerariae Lobatae Radix (PLR)* has a long history of use as a traditional Chinese medicine. It has a variety of biological activities, including the ability to lower blood pressure and blood lipids (Salehi et al., 2019; Sham et al., 2014; Song et al., 2014; Zhang, 2019). As an edible traditional Chinese medicine, it is widely used in the development of drugs, health foods, and general food products (Zhang et al., 2020). A few studies of its general toxicity (Jo et al., 2013; Seong et al., 2006; Song et al., 2020; Tam et al., 2009) have shown that *PLR* has a relatively high safety. However, there is some evidence that *PLR* and its main components may have safety risks in some populations. The long‐term and high‐dose consumption of *PLR* and its main components in specific populations may have toxic effects on the liver and reproductive system (Chen & Chan, 2009; Huang et al., 2016; Santosh et al., 2010). Long‐term administration of *PLR* (100 mg/kg) might affect the mating and reproductive efficiency of adult female mice (Jaroenporn et al., 2007). *PLR* extract could increase the serum alanine aminotransferase, aspartate aminotransferase in mice, and made liver histopathological changes which indicated that *PLR* extract has hepatotoxicity (Wang et al., 2015).

The main components of *PLR* are isoflavones, which belong to phytoestrogens, including 3′‐hydroxy puerarin, puerarin, 3′‐methyoxy‐puerarin, pueraria glycoside 2, mirificin, and daidzin (Song et al., 2014; Wu et al., 2018). Phytoestrogens often have estrogen‐like or anti‐estrogen‐like effects. However, the precise safety and adverse effects of the long‐term use of *PLR* and its products in specific populations are not well‐established (Jaroenporn et al., 2006; Jefferson et al., 2012, 2020; Kakehashi et al., 2016; Suchinda et al., 2004). Adolescence is the first period of reproductive ability. The reproductive organs during puberty are basically mature, at the peak of growth and development, hormone levels exhibit a rapid rise, and hypothalamic–pituitary–gonad axis development is imperfect. Abnormal estrogen levels may result in female precocity or delayed male development.

Metabolomics is an integral approach in systems biology and is widely used in many fields of research to study relationships between metabolites and physiological and pathological changes (Han et al., 2018; Lindon & Nicholson, 2008; Patti et al., 2012; Rosato et al., 2018). The approach is based on the high‐throughput detection of small molecule metabolites in organisms and statistical analyses of multivariate data. Finally, pathway analyses are used to understand the biological significance of differential metabolites (Barnes et al., 2016; Bartel et al., 2013; Zhu et al., 2018).

In this study, pharmacological experiments combined with serum metabolomics technology were used to evaluate the safety of high‐dose *PLR* in adolescent *SD* rats. These results improve our understanding of the safety of *PLR* and provide a scientific basis for the use of *PLR* (Yan et al., 2017; Yu et al., 2019).

## 2 MATERIALS AND METHODS

### 2.1 Medicinal materials and reagents

Pueraria Lobata Radix (Shanxi Jinshitianrun Geye Co., Ltd.; batch number: 20190228) was identified as the dry root of the leguminous plant *Pueraria lobata* (Willd) Ohwi by the Jiangxi University of Traditional Chinese Medicine Appraisal Department. The Rat Aromatase ELISA Kit was obtained from Shanghai Yuchun Biotechnology Co., Ltd. (batch number: 201907). Additionally, 10% neutral formalin fixative (Beijing Leigen Biotechnology Co., Ltd.; batch number: 0412A19), LC‐MS‐grade formic acid (Beijing Bailingwei Technology Co., Ltd.; batch number: L590S65), methanol and acetonitrile (chromatographic grade; Merck Group Co., Ltd.), and distilled water (Guangzhou Watsons Food and Beverage Co., Ltd.) were used.

### 2.2 Instruments

The instruments were as follows: a fully automatic blood cell analyzer (Mindray Medical International Co., Ltd.; model: BC‐5380), fully automatic biochemical analyzer (Beckman, Brea, CA, USA; model: AU480), automatic radioimmunoassay (Siemens; model: centuarxp), Ultrapure water meter (Nanjing EPED Technology Development Co., Ltd.; model: EPED‐ESL‐10), inverted microscope (Nikon, Tokyo, Japan; model: TS100), high‐speed refrigerated centrifuge (SIGMA; model: 3‐18K), vortex mixer (IKA Instruments Co., Ltd.; model: VORTEX GENIUS3), and the UltiMate 3000 UHPLC System and LTQ ORBITRAP VELOS PRO high‐resolution mass spectrometer (Thermo Fisher).

### 2.3 Extraction of *PLR*

Dried PLR (15 kg) was used for two extraction steps with a 10‐fold volume of double‐distilled water, 2 hr each time. After filtering, the two extracts were combined and concentrated under reduced pressure.

### 2.4 Experimental animals

A total of 60 SPF *SD* rats, 4–5 weeks old and weighing 70–90 g, were obtained from the Experimental Animal Center of Jiangxi University of Traditional Chinese Medicine (Experimental Animal License Number: SCXK (Gan) 2018‐0003). The feeding conditions were 20–23°C, humidity 45%–55%, alternate dark cycle for 12 hr (license number SYXK (Gan) 2018‐0004). Rats were provided free access to a standard diet and water. Sterilized granular feeds were provided by Wanqian Jiaxing Co., Ltd. The study was approved by the Experimental Animal Ethics Sub‐Committee of the Academic Committee of Jiangxi University of Traditional Chinese Medicine and complies with the animal research guidelines of the China Ethics Committee.

After 1 week of acclimatization, 60 rats were randomly divided into three groups, a blank control group, low‐dose group, and high‐dose group, with 20 rats per group (10 males and 10 females). The *PLR* water concentrate was administered intragastrically. The low‐dose and high‐dose groups were treated with 50 times and 100 times the daily dose of *PLR* tablets prescribed by the pharmacopoeia (i.e., 12.5 g/kg and 25 g/kg), and animals in the blank control group were given an equal volume of purified water. For 30 days of continuous administration, animal activity and growth were observed daily, body weight was recorded once a week, and the dosage was adjusted according to weight. Water was freely available during the study period.

### 2.5 Acquisition and processing of experimental samples

After fasting overnight on the day of the last dose, blood samples and organs were collected from male rats (day 31). Female rats were tested for vaginal exfoliated cell smears at 7:00 in the morning for several days after the final dose. The blood and organs of female rats in the diestrus period (after regular administration and fasting overnight) were collected in the afternoon on the next day. Part of the whole blood was centrifuged at 845 g for 10 min, and the serum samples were stored at −80°C (Schaalan et al., 2018; Sharma et al., 2014).

### 2.6 Organ wet weight and organ coefficients

After dislocating the cervical vertebrae, the liver, kidney, breast, uterus, ovary, testis, epididymis, and other organs and tissues were immediately dissected. The surrounding connective and fatty tissues were carefully removed and washed with normal saline. The organs were weighed, and the organ coefficient was estimated as the ratio of the wet weight of the organ to the body weight (Chen et al., 2016; Chen, Gong, et al., 2016).

### 2.7 Blood indicators and serum biochemical tests

A fully automatic blood cell analyzer was used to detect HB (hemoglobin), RBC (red blood cells), WBC (white blood cells), PLT (platelets), and other routine indicators. A fully automatic blood biochemical analyzer was used to detect AST (Aspartate aminotransferase), ALT (Aspartate aminotransferase), ALP (Alkaline phosphatase), DBIL (direct bilirubin), TBIL (total bilirubin), TBA (total bile acid), ADA (adenosine deaminase), AFU (α‐L‐fucosidase), ALB (albumin), TP (total protein), BUN (blood urea nitrogen), Cr (Creatinine), UA (Uric acid), and other indicators (Chen, Chen, et al., 2016; Chen, Gong, et al., 2016; Yu et al., 2017).

### 2.8 Determination of estradiol and testosterone in the serum

The estradiol (E2) and testosterone (T) levels in rat serum were measured by radioimmunoassays (Lai et al., 2009). Because female rats have different hormone levels depending on the phase of the estrous cycle, rats in the same phase were selected for the detection of hormone levels. In this experiment, hormone levels were determined during diestrus.

### 2.9 Histopathological examination

After dissection, gross visual inspection showed no obvious tissue abnormalities. The liver, kidney, breast, testis, epididymis, ovary, and uterus tissues of the high‐dose group were fixed, dehydrated, embedded, sectioned, and hematoxylin and eosin (HE)‐stained. Sections were examined under a microscope at 40× and 100× (Liu et al., 2014; Zhao et al., 2020).

### 2.10 Serum metabolomics analysis

#### 2.10.1 Pretreatment of serum samples

The serum samples were thawed at 4°C, 100‐μl aliquots were obtained, and 200 μl of methanol was added. After vortexing for 3 min, samples were centrifuged for 10 min (4°C, 18407 g). Then, 100 µl of the supernatant was added to an EP tube and blow dried with nitrogen. The residue was supplemented with 100 µl of methanol, vortexed for 1 min, and centrifuged for 10 min (4°C, 14,000 rpm). The supernatant was added to sample vials for testing. For QC sample preparation, 10‐µl aliquots of all serum samples were mixed uniformly and treated according to the above serum pretreatment method (Cheng et al., 2020).

#### 2.10.2 Chromatographic conditions

Ultra‐high liquid chromatography (UltiMate 3000 UPLC System, Thermo Scientific) was performed using the ACE UltraCore C18 Column (100 mm × 2.1 mm, 2.5 μm). Mobile phase A was 0.1% formic acid water and B was acetonitrile. The flow rate was 0.30 ml/min, and column temperature was 30°C; gradient elution and elution conditions are shown in Table 1.

**Table 1:** Chromatographic gradient elution conditions

time (min)	A (%)	B (%)
0	95	5
1	95	5
5	44	56
14	26	74
19	0	100
19.1	95	5
22	95	5

#### 2.10.3 Mass spectrometry conditions

The mass spectrometer was operated in positive ion mode with a heated electrospray ion source (HESI), ion source temperature 350°C, capillary temperature 320°C, sheath gas flow rate 35 units, auxiliary gas flow rate 10 units, spray voltage 4 kV, capillary voltage 35 V, and tube lens voltage 110 V. For the first full scan, the resolution was set to 30,000, and the scan range was m/z 100–1000; for the second level, dynamic data‐dependent scanning (DDS) was used to select the top three peaks from the previous level by collision‐induced dissociation (CID), fragment scanning, and detection with an ion trap dynode.

For negative ion mode, the following parameter settings were used: heated electrospray ion source (HESI), ion source temperature 300°C, capillary temperature 320°C, sheath gas flow rate 35 units, auxiliary gas flow rate 10 units, spray voltage 3.6 kV, capillary voltage 35 V, and tube lens voltage 110 V. For the first full scan, the resolution was set to 30,000 and the scan range was m/z 100–1000. For the second level, DDS was used to select the top six peaks of the previous level for CID, fragment scanning, and detection with an ion trap dynode.

### 2.11 Data analysis and processing

By LTQ ORBITRAP VELOS PRO mass spectrometry, positive and negative ion modes were used to collect sample data. The raw data were preprocessed using SIEVE, including deconvolution, peak identification, peak alignment, baseline correction, peak area normalization, and other data preprocessing. The processed data matrix was filtered using a threshold frequency of 80% and the filtered data matrix was imported into simca‐p14.0 for multivariate statistical analyses, including a principal component analysis (PCA) and orthogonal partial least square discriminant analysis (OPLS‐DA) (Bijlsma et al., 2006; Li et al., 2020; Su et al., 2020). The OPLS‐DA model was used to screen compounds with VIP > 1, and these data were imported into SPSS for *t* tests to identify compounds with significant differences at *p* < .05. Compounds with both VIP > 1 and *p* < .05 were treated as differential metabolites. The structure of differential metabolites was confirmed by secondary mass spectrometry of differential metabolites and related databases, such as Metlin, Human Metabolite Database, and MassBank. Differential metabolite information was imported into the metaboanalyst3.0 database for a metabolic pathway analysis (Beyoğlu & Idle, 2020; Han et al., 2019; Wang et al., 2019; Xing et al., 2017).

The experimental data were analyzed using SPSS17.0. Measurement data are expressed as means ± standard deviation. Groups were compared using *t* tests to identify significant (*p* < .05) and extremely significant differences (*p* < .01).

## 3 RESULTS

### 3.1 Main chemical components of *PLR* water extract

**Figure 1:**
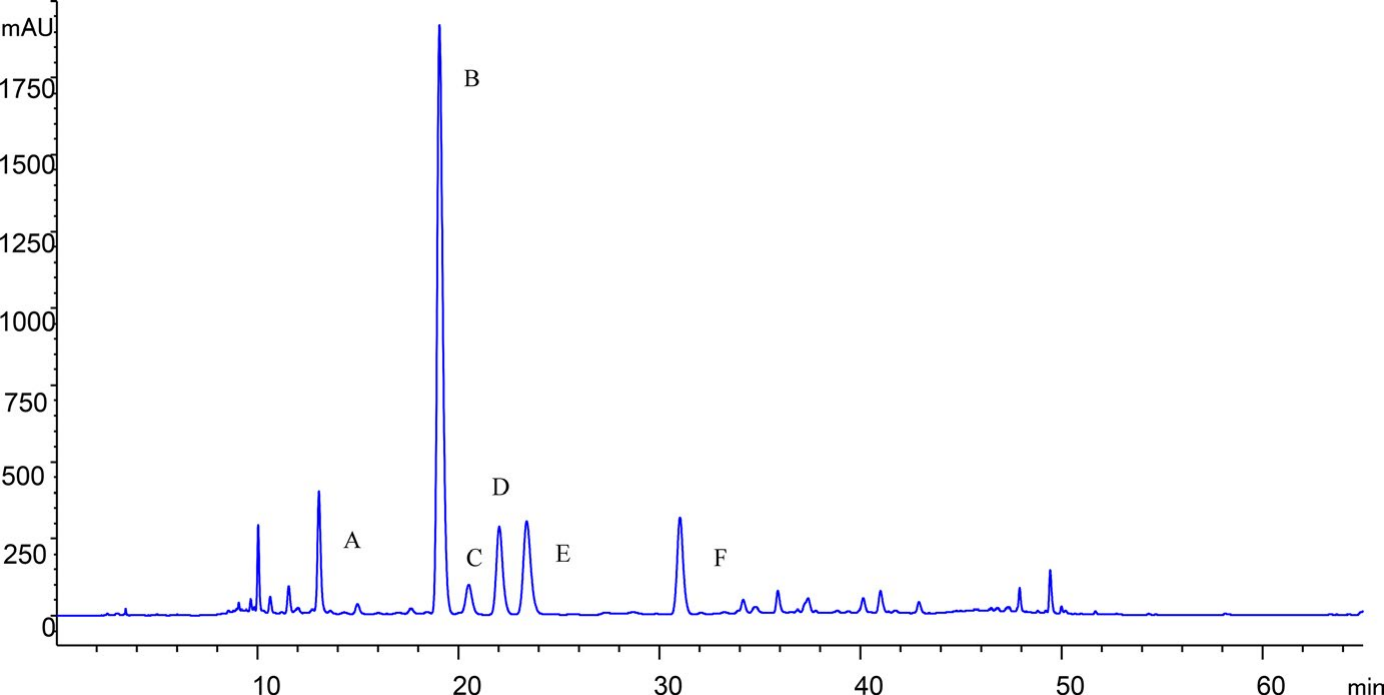
Chromatogram of *Puerariae Lobatae Radix* water extract. A. 3′‐hydroxy Puerarin, b. Puerarin, C. 3′‐Methyoxy‐puerarin, D. Pueraria glycoside 2, E. Mirificin, F. Daidzin

The components of *PLR* water extract included 3′‐hydroxy puerarin, puerarin, 3′‐methyoxy‐puerarin, pueraria glycoside 2, mirificin, and daidzin, among which the puerarin content was highest (Figure 1).

### 3.2 General performance of rats after *PLR* administration

During the 30‐day administration period, male and female rats of each group (low‐dose, high‐dose, and blank control) did not show signs of a refusal to eat. No deaths were observed. Furthermore, rats showed a normal mental state and active growth, with thick and shiny coats.

### 3.3 Effects of *PLR* on body weight and organ coefficients in rats

There were no statistically significant differences in weekly weight gain and total weight gain in male and female rats between the treatment groups (low and high dose) and the blank control group (*p* > .05) (Figure S1). A high dose of *PLR* had no significant effect on the weight of male and female rats.

The absolute weights (Figure S2) and organ coefficients (Figure S3) of the liver, kidney, uterus, ovary, testis, and epididymis in the low‐dose group and the high‐dose group did not differ significantly from those in the blank control group (*p* > .05). These results indicated that *PLR* had no effect on the absolute weight and organ coefficients of male and female rats.

### 3.4 Effects of *PLR* on blood indexes in rats

There were no statistically significant differences in the number of WBC, the number of RBC, the concentration of HB, and the number of PLT among groups (*p* > .05) (Figure S4). These results indicated that *PLR* had no adverse effects on biochemical indexes in rats.

### 3.5 Effects of *PLR* on serum biochemical indexes related to liver function

As shown in Tables 2 and 3, compared with the blank control group, the ALP, ALT, and TP contents of female rats in the low‐dose group and high‐dose group were significantly different. In particular, the ALP and TP levels in female *SD* rats in the low‐dose group and high‐dose group were very significantly higher (*p* < .01), ALT levels in female rats in the low‐dose group were significantly different (*p* < .05), and ALT levels in female rats in the high‐dose group were very significantly different (*p* < .01) than those in the control group. Compared with the blank control group, there were no significant differences in biochemical indicators of liver function, including ALP, ALT, TBIL, DBIL, TBA, AST, TP, LP (a), ADA, AFU, and ALB, in male rats in the low‐dose group and the high‐dose group (*p* > .05). These results indicated that *PLR* did not influence biochemical indexes of liver function in male rats but altered the levels of ALP, ALT, and TP in female rats.

**Table 2:** Effects of *PLR* on serum biochemical indexes of liver function in rats ($\bar{X}$ ± S, *n* = 10)

Sex	Group	ALP (U/L)	ALT (U/L)	DBIL (μmol/L)	TBA (μmol/L)	TBIL (μmol/L)	AST (U/L)
female	Blank control	78.92 ± 12.77	25.44 ± 5.08	1.83 ± 0.16	20.73 ± 7.38	2.19 ± 0.12	114.30 ± 13.47
	Low does	109.27 ± 20.97**	43.24 ± 21.31*	1.86 ± 0.15	31.85 ± 14.22	2.12 ± 0.44	156.25 ± 81.10
	High does	101.04 ± 15.25**	47.79 ± 21.86**	1.94 ± 0.10	27.30 ± 10.54	2.34 ± 0.40	152.14 ± 131.32
male	Blank control	122.08 ± 19.71	37.81 ± 9.61	1.94 ± 0.17	19.54 ± 5.62	2.18 ± 0.27	139.96 ± 35.40
	Low does	137.88 ± 23.23	38.70 ± 6.87	1.82 ± 0.34	17.22 ± 5.08	2.13 ± 0.21	114.27 ± 24.53
	High does	140.49 ± 32.82	48.33 ± 10.22	1.85 ± 0.29	24.75 ± 4.81	1.95 ± 0.67	144.32 ± 28.92

* Means *p* < .05 compared with blank control group.

** Means *p* < .01 compared with blank control group

**Table 3:** Effects of *PLR* on serum biochemical indexes of liver function in rats ($\bar{X}$ ± S, *n* = 10)

Sex	Group	TP (g/L)	Lp(a) (mmol/L)	ADA (μmol/L)	AFU (u/g)	ALB (g/L)
female	Blank control	69.08 ± 3.92	50.62 ± 5.91	24.37 ± 25.10	2.93 ± 0.90	42.44 ± 2.59
	Low does	75.36 ± 3.31**	55.96 ± 4.80	23.28 ± 29.62	3.86 ± 1.00	44.38 ± 3.22
	High does	75.96 ± 5.94**	55.17 ± 4.19	25.21 ± 25.07	3.84 ± 1.73	45.12 ± 4.90
male	Blank control	77.60 ± 6.03	54.49 ± 3.92	9.09 ± 5.39	2.66 ± 1.36	45.96 ± 4.29
	Low does	73.30 ± 3.38	57.51 ± 3.71	16.01 ± 14.24	3.29 ± 1.51	44.61 ± 3.37
	High does	74.91 ± 7.58	52.07 ± 6.31	9.53 ± 4.95	3.21 ± 1.36	44.86 ± 5.52

^*^Means *p* < .05 compared with blank control group.

** Means *p* < .01 compared with blank control group.

### 3.6 Effects of *PLR* on serum biochemical markers of renal function

As shown in Table 4, compared with the blank control group, there were no statistically significant differences in the serum biochemical indexes BUN, Cr, and UA in the low‐dose group and the high‐dose group (*p* > .05). These results showed that *PLR* had no adverse effects on biochemical markers of renal function in male and female rats.

**Table 4:** Effects of *PLR* on serum biochemical indexes of renal function in rats ($\bar{X}$ ± S, *n* = 10)

Sex	Group	BUN (mmol/L)	Cr (μmol/L)	UA (μmol/L)
female	Blank control	6.73 ± 1.30	25.78 ± 2.99	103.06 ± 13.31
	Low does	6.65 ± 2.11	27.58 ± 2.83	95.32 ± 12.38
	High does	7.47 ± 1.55	23.99 ± 4.00	96.41 ± 12.27
male	Blank control	7.15 ± 1.35	20.84 ± 3.45	95.84 ± 9.58
	Low does	6.01 ± 1.24	20.12 ± 1.98	93.50 ± 16.48
	High does	7.83 ± 1.25	21.47 ± 4.84	113.43 ± 22.51

^*^Means *p* < .05 compared with blank control group.

^**^Means *p* < .01 compared with blank control group.

### 3.7 Effects of *PLR* on serum estradiol and testosterone in rats

As shown in Table 5, compared with the blank control group, E2 levels in female rats in the low‐dose group and the high‐dose group and in male rats in the high‐dose group were significantly different (*p* < .01). The T levels in female and male rats in the low‐dose group and the high‐dose group did not differ significantly among groups (*p* > .05). Based on these results, *PLR* significantly increases levels of the sex hormone E2 in male and female rats.

**Table 5:** Effects of *PLR* on sex hormone indexes in rats ($\bar{X}$ ± S, *n* = 10)

Sex	Group	Estradiol (pg/ml)	Testosterone (ng/dl)
female	Blank control	25.88 ± 3.11	15.00 ± 3.93
	Low does	41.95 ± 9.64**	13.66 ± 3.00
	High does	45.61 ± 10.61**	14.66 ± 5.31
male	Blank control	16.15 ± 2.98	321.25 ± 240.22
	Low does	18.64 ± 3.06	266.77 ± 197.61
	High does	26.97 ± 3.29**	435.66 ± 409.55

^*^Means *p* < .05 compared with blank control group.

** Means *p* < .01 compared with blank control group.

### 3.8 Histopathological examination

**Figure 2:**
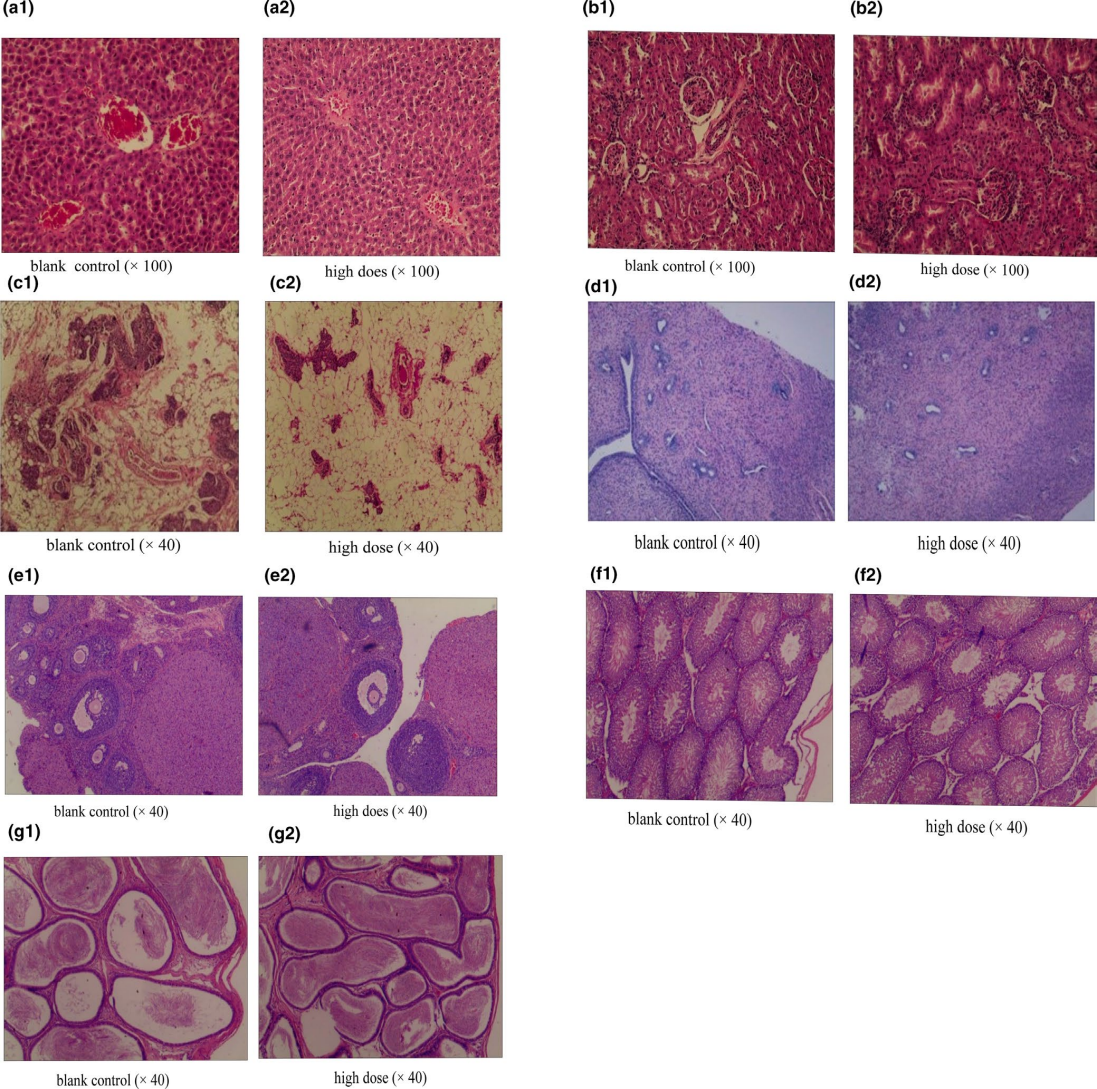
Effects of *Puerariae Lobatae Radix* on histopathology in rats. a. liver, b. kidney, c. breast, d. uterus, e. ovary, f. testis, and g. epididymis

With respect to gross anatomy, no significant abnormalities were observed in rat organs of the low‐dose group, high‐dose group, or blank control group. A light microscopy analysis showed that the liver, kidney, breast, uterus, ovary, testis, and epididymis did not differ with respect to histopathological features between the high‐dose group and the blank control group, as shown in Figure 2.

### 3.9 UPLC‐MS total ion chromatograms

**Figure 3:**
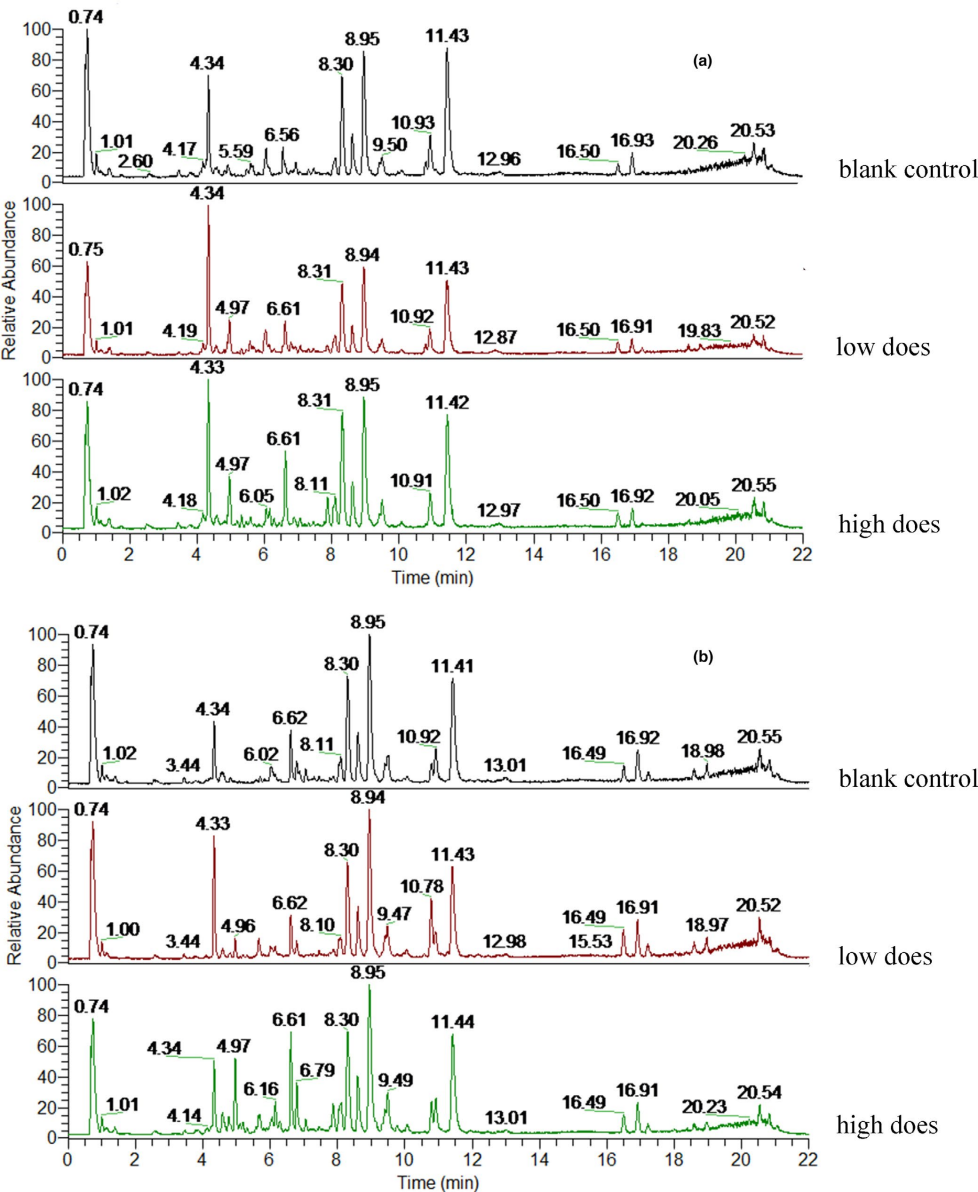
Total ions of female *SD* rats (a is positive, and b is negative)

**Figure 4:**
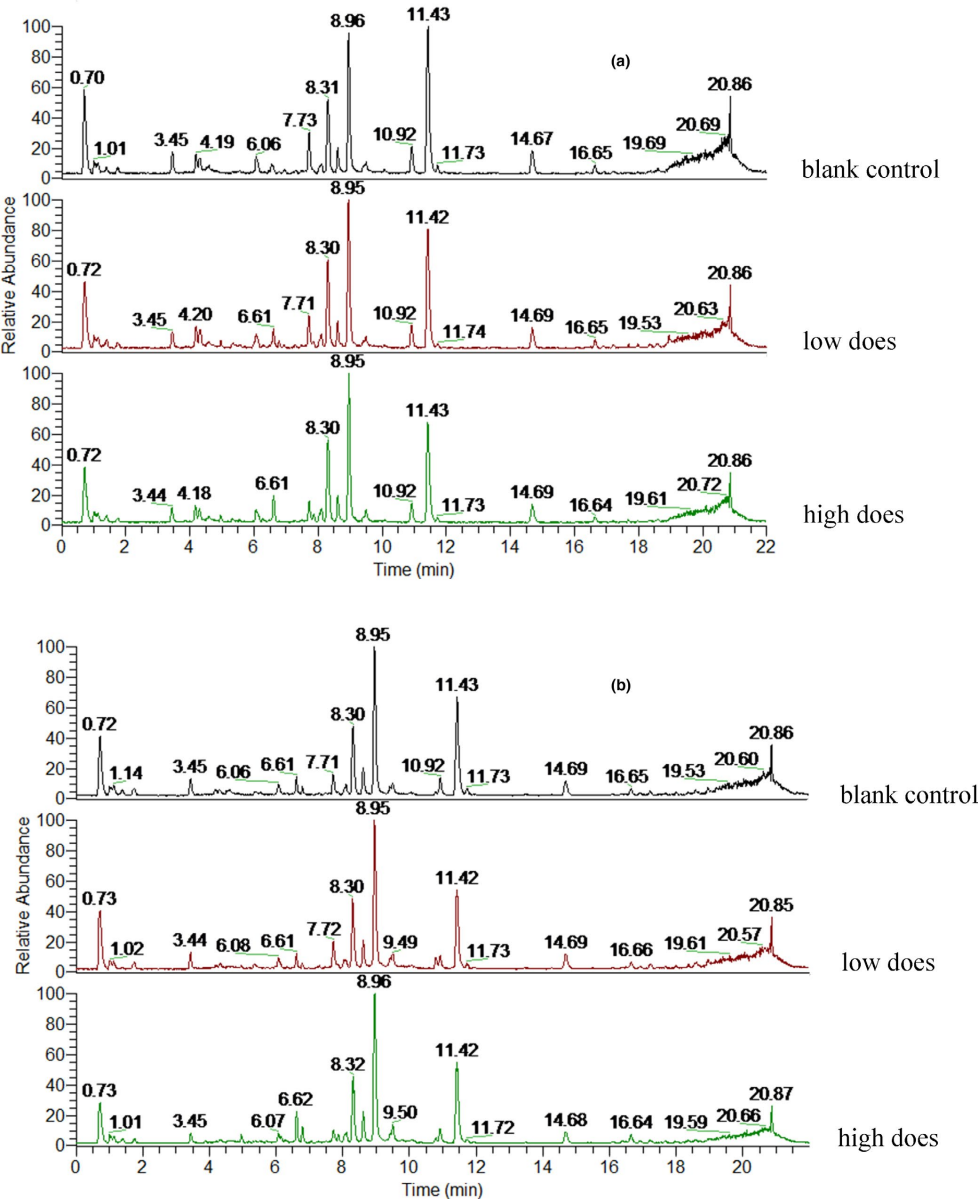
Total ions of male *SD* rats (a is positive. and b is negative)

To obtain as much compound information as possible, positive and negative ion modes were used for data collection and representative differential metabolites were obtained. As shown in Figures 3 and 4, the serum total ion chromatograms (TIC) for male and female rats in different groups were essentially the same, with some differences in intensity.

#### 3.9.1 Principal component analysis

**Figure 5:**
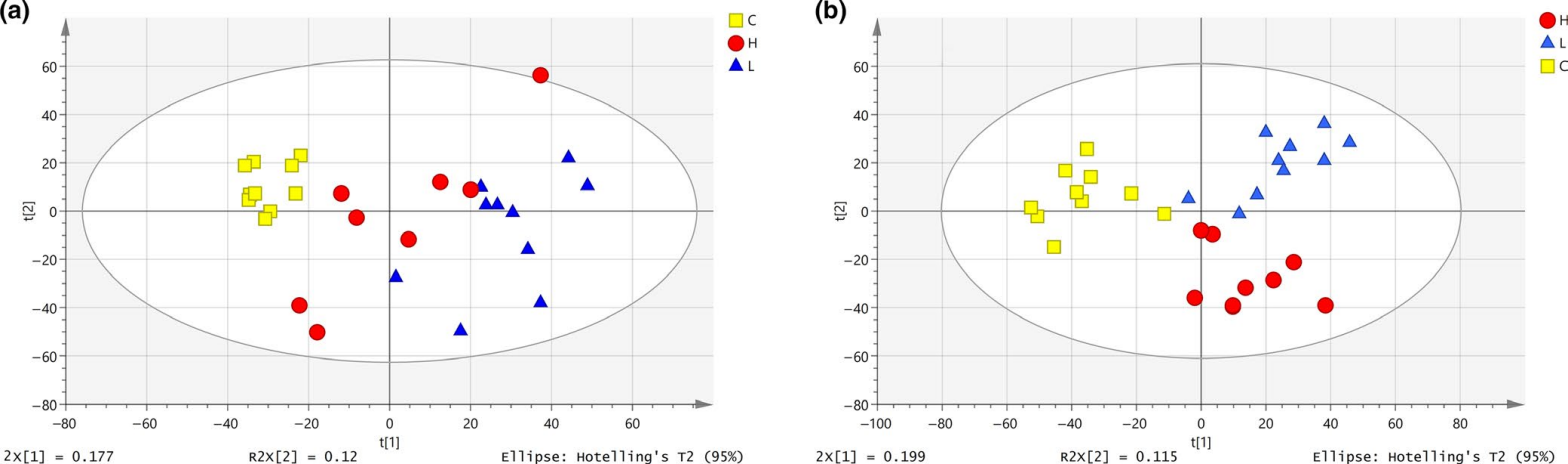
PCA score plot of rats. a. female rats, b. male rats

Metabolic profiles of serum samples were obtained for each group. In the PCA score plot for female rats fed *PLR* for 30 days, five principal components showed a $R_{X}^{2}=0.501$, *Q* ^2^ = 0.148. As shown in Figure 5a, there was clear separation among female rats in the *PLR* low‐dose group (L), high‐dose group (H), and blank control group (C). Female rats in the blank control group showed smaller differences and greater aggregation, while the high‐dose and low‐dose groups showed greater variation and more discrete aggregation, suggesting that the effect of *PLR* on individual female rats was highly variable. In the PCA score plot for male rats fed *PLR* for 30 days, five principal components showed a cumulative $R_{X}^{2}=0.532,$ *Q* ^2^ = 0.231. As shown in Figure 5b, male rats showed complete separation among groups. The degree of aggregation of male rats in different groups was similar. PCA could be used for a preliminary assessment of the metabolite profiles of rats in different groups; however, owing to the variation within groups (and inability to highlight the differences between groups), further OPLS‐DA was needed.

#### 3.9.2 OPLS‐DA of female rats in different groups

**Figure 6:**
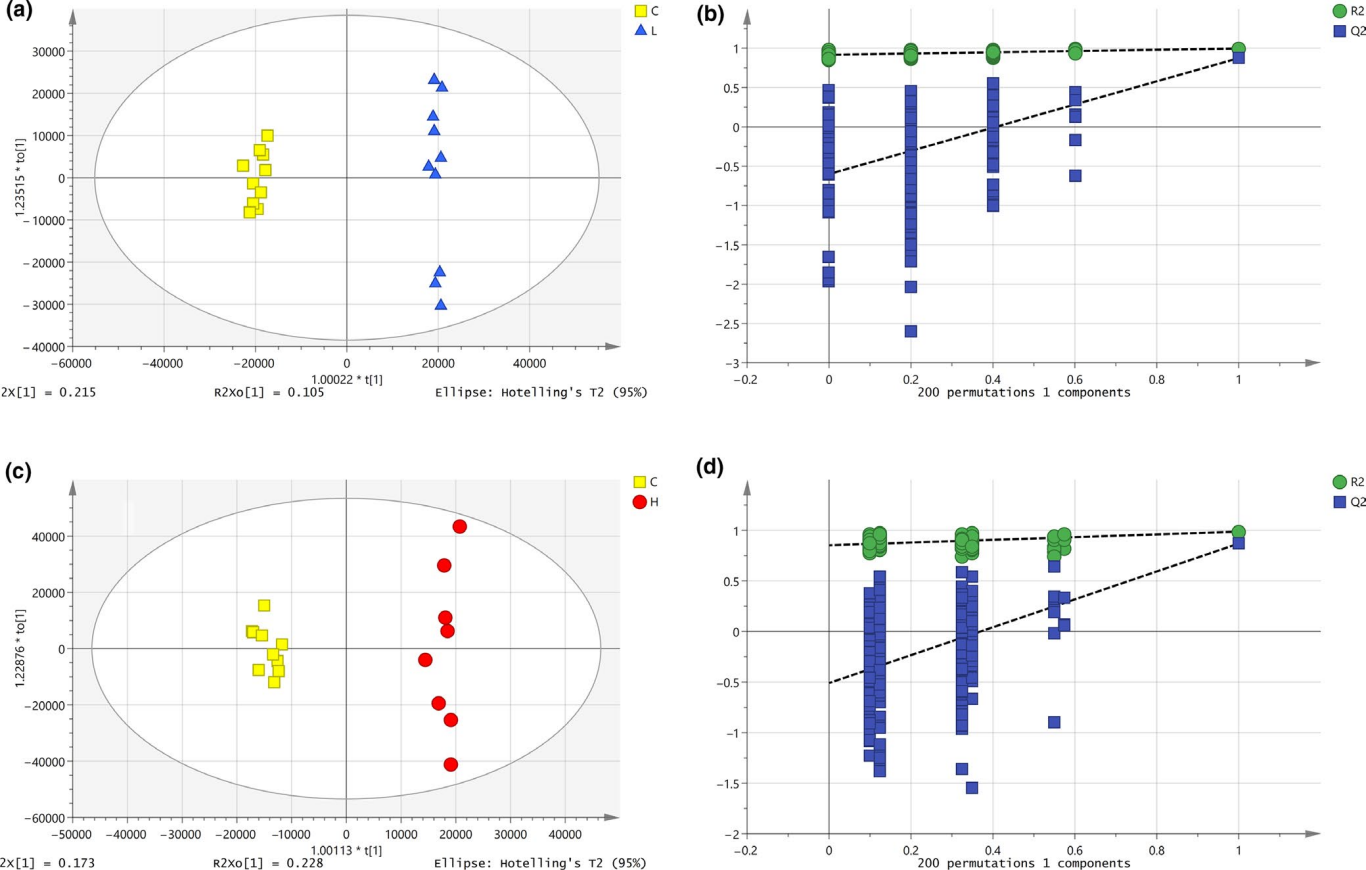
OPLS‐DA analysis and Permutation test of female. a. OPLS‐DA score plot of L and C groups, b. Permutation test of L and C groups, c. OPLS‐DA score plot of H and C groups, and d. Permutation test of H and C groups

As shown in Figure 6a, an OPLS‐DA model for female rats in the low‐dose group (L) and the blank control group (C) was established. The samples of female rats in the low‐dose group and the blank control group were completely spatially separated, with = $R_{X}^{2}$0.549, $R_{Y}^{2}$ = 0.996, *Q* ^2^ = 0.874, indicating that the model had good predictive ability. A permutation test repeated 200 times, as shown in Figure 6b, showed that as *Y* variables increased, *R* ^2^ and *Q* ^2^ gradually declined, indicated that the model was highly robust and there was no overfitting.

As shown in Figure 6c, an OPLS‐DA model for female rats in the high‐dose group (H) and the blank control group (C) was established. Samples of female rats in the high‐dose group and the blank control group were completely spatially separated, with = $R_{X}^{2}$0.584, $R_{Y}^{2}$= 0.987, *Q* ^2^ = 0.870, indicating that the model had good predictive ability. A permutation test repeated 200 times, as shown in Figure 6d, showed that as *Y* variables increased, *R* ^2^ and *Q* ^2^ gradually declined, indicating that the model was highly robust and there was no overfitting.

#### 3.9.3 OPLS‐DA of male rats in different groups

**Figure 7:**
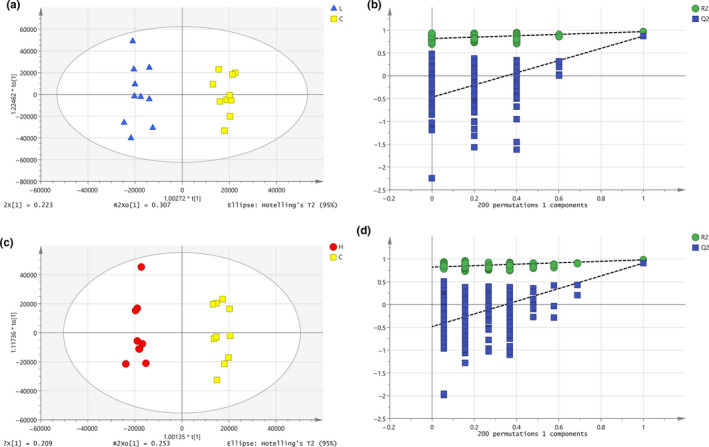
OPLS‐DA analysis and Permutation test of male. a. OPLS‐DA score plot of L and C groups, b. Permutation test of L and C groups, c. OPLS‐DA score plot of H and C groups, and d. Permutation test of H and C groups

As shown in Figure 7a, an OPLS‐DA model for male *SD* rats in the low‐dose group (L) and the blank control group (C) was established. The samples of male *SD* rats in the low‐dose group and the blank control group were completely spatially separated, with = $R_{X}^{2}$0.620, $R_{Y}^{2}$= 0.969, *Q* ^2^ = 0.871, indicating that the model had good predictive ability. A permutation test repeated 200 times, as shown in Figure 7b, showed that as *Y* variables increased, *R* ^2^ and *Q* ^2^ gradually declined, indicated that the model was highly robust and there was no overfitting.

As shown in Figure 7c, an OPLS‐DA model of male rats in the high‐dose group (H) and the blank control group (C) was established. The samples of male rats in the high‐dose group and the blank control group were completely spatially separated, with = $R_{X}^{2}$0.560, $R_{Y}^{2}$ = 0.980, *Q* ^2^ = 0.908, indicating that the model had good predictive ability. A permutation test repeated 200 times, as shown in Figure 7d, showed that as *Y* variables increased, *R* ^2^ and *Q* ^2^ gradually declined, indicating that the model was highly robust and there was no overfitting.

#### 3.9.4 Differential metabolites between the low‐dose group and blank control group in female rats

Using VIP > 1 and *p* < .05 as thresholds, 12 differential metabolites were identified. As shown in Table 6, compared with the blank control group, the levels of eight metabolites (i.e., PC(16:0/0:0)[U], glycocholate, docosahexaenoic acid ethyl ester, deoxycholic acid, 6‐ethoxy‐2‐mercaptobenzothiazole, cortisone, lathosterol, and Ala Asn Ile Lys) were significantly up‐regulated, and levels of four metabolites (i.e., carnitine, leukotriene A4 methyl ester, tryptophan, and Glu Met Trp) were significantly down‐regulated in the low‐dose group.

**Table 6:** Serum differential metabolites of female rats between low‐dose group and blank control group

No.	m/z	Time	Metabolite	VIP	*p*	FC
1	496.3391	8.95	PC(16:0/0:0)[U]	16.80	0.016	1.24
2	464.3012	6.01	Glycocholate	4.82	0.009	3.70
3	357.2786	6.79	Docosahexaenoic Acid ethyl ester	4.46	0.022	2.61
4	162.1125	0.72	Carnitine	4.13	0.000	0.58
5	391.2854	6.78	Deoxycholic acid	3.00	0.040	2.00
6	212.0201	6.12	6‐Ethoxy‐2‐mercaptobenzothiazole	2.78	0.000	2.18
7	359.1902	7.93	Cortisone	2.71	0.008	3.27
8	369.3513	20.84	Lathosterol	2.24	0.000	1.85
9	333.2423	6.54	Leukotriene A4 methyl ester	1.67	0.005	0.57
10	203.0828	3.42	Tryptophan	1.59	0.001	0.67
11	443.2579	6.60	Ala Asn Ile Lys	1.49	0.003	2.94
12	465.1800	7.61	Glu Met Trp	1.05	0.001	0.76

#### 3.9.5 Differential metabolites between the high‐dose group and blank control group in female rats

Using VIP > 1 and *p* < .05 as thresholds, 9 differential metabolites were identified. As shown in Table 7, compared with the blank control group, the levels of six metabolites (i.e., docosahexaenoic acid ethyl ester, deoxycholic acid, isohyodeoxycholic acid, cortisone, Ala Asn Ile Lys, and lathosterol) were significantly up‐regulated, and the levels of three metabolites (i.e., carnitine, leukotriene A4 methyl ester, and tryptophan) were significantly down‐regulated in the high‐dose group.

**Table 7:** Serum differential metabolites of female rats between high‐dose group and blank control group

No.	m/z	Time	Metabolite	VIP	*p*	FC
1	357.2786	6.79	Docosahexaenoic Acid ethyl ester	4.84	0.033	2.38
2	162.1125	0.72	Carnitine	4.66	0.001	0.57
3	391.2854	6.78	Deoxycholic acid	3.89	0.024	2.26
4	391.2864	8.11	Isohyodeoxycholic acid	3.87	0.014	2.17
5	359.1902	7.93	Cortisone	3.81	0.002	4.13
6	333.2423	6.54	Leukotriene A4 methyl ester	2.44	0.000	0.37
7	203.0828	3.42	TRYPTOPHAN	1.68	0.010	0.72
8	443.2579	6.60	Ala Asn Ile Lys	1.54	0.012	2.72
9	369.3513	20.84	Lathosterol	1.30	0.048	1.35

#### 3.9.6 Cluster analysis of differential metabolites in female rats

**Figure 8:**
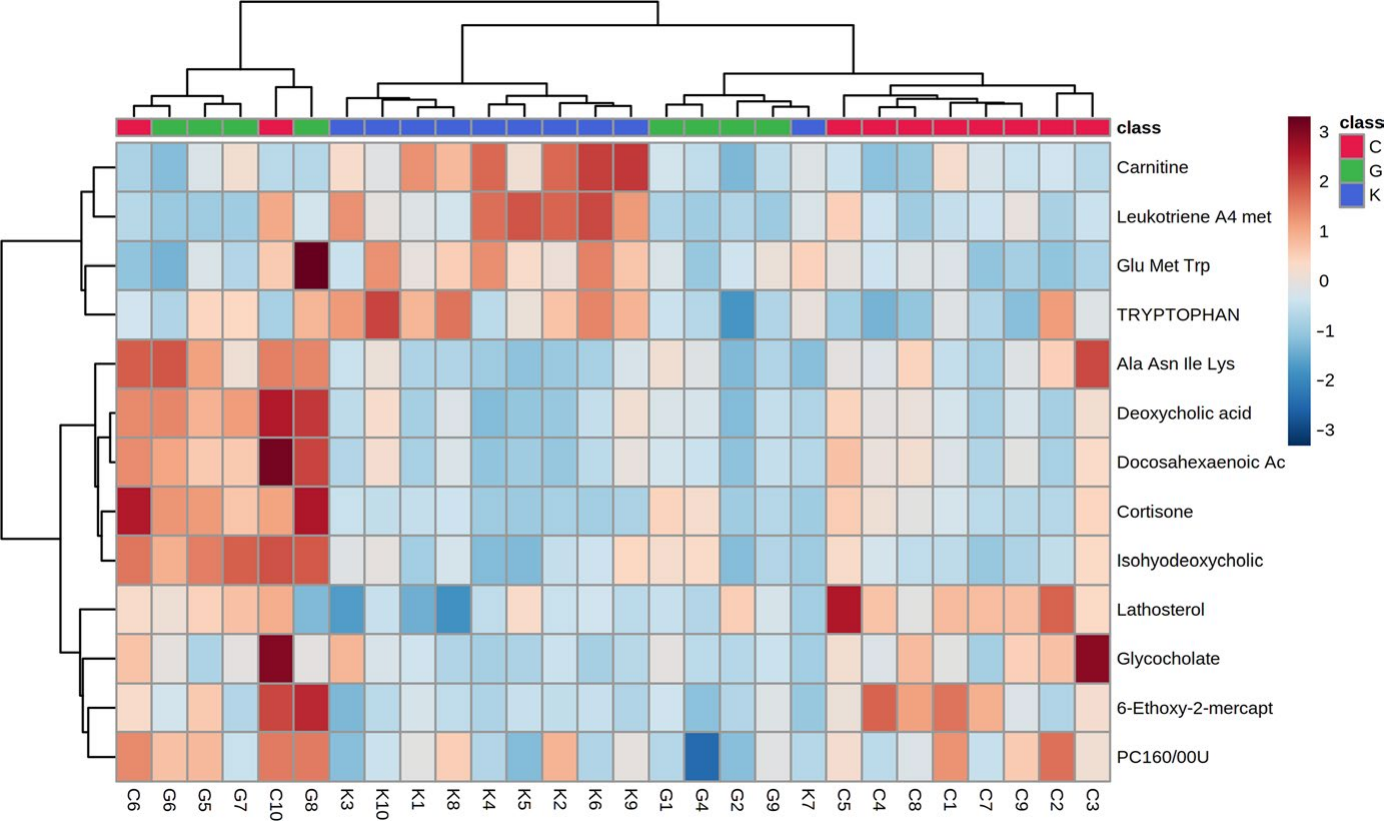
Heat map of serum differential metabolites of female rats in different dose groups

As shown in Tables 6 and 7, differential metabolites between the low‐dose group and the high‐dose group were almost the same as those in comparisons between these groups and the blank control group. For an intuitive representation of metabolite differences, a heat map was used to visualize hierarchical clustering results for 13 differential metabolites among the low‐dose group, high‐dose group, and blank control group. As shown in Figure 8, the differential metabolites in the blank control group and the treatment groups (low‐dose group and high‐dose group) showed clear separation. (where colors from red to blue represent the relative content of the substance in the corresponding serum sample, from high to low).

#### 3.9.7 Different metabolites between the low‐dose group and blank control group in male rats

Using VIP > 1 and *p* < .05 as thresholds, 7 differential metabolites were identified. As shown in Table 8, compared with the blank control group, the levels of seven metabolites (i.e., PC(16:0/0:0)[U], phenylalanine, cortisone, glycerophospho‐*N*‐palmitoyl ethanolamine, 1‐acetylindole, Ser Tyr Arg, and Arg Phe Arg) were significantly up‐regulated in males of the low‐dose group.

**Table 8:** Serum differential metabolites of male rats between low‐dose group and blank control group

No.	m/z	Time	Metabolite	VIP	*p*	FC
1	496.3391	8.95	PC(16:0/0:0)[U]	24.89	0.019	1.33
2	166.0861	1.72	Phenylalanine	2.50	0.033	1.11
3	359.1902	7.93	Cortisone	2.47	0.007	1.79
4	454.2927	8.87	Glycerophospho‐N‐Palmitoyl Ethanolamine	1.89	0.002	1.65
5	160.0758	1.35	1‐Acetylindole	1.31	0.007	1.39
6	425.2143	14.23	Ser Tyr Arg	1.14	0.000	1.25
7	478.2924	8.24	Arg Phe Arg	1.05	0.002	1.78

#### 3.9.8 Different metabolites between the high‐dose group and blank control group in male rats

Using VIP > 1 and *p* < .05 as thresholds, 15 differential metabolites were identified. As shown in Table 9, compared with the blank control group, the levels of 13 metabolites (i.e., docosahexaenoic acid ethyl ester, deoxycholic acid, cortisone, phenylalanine, isohyodeoxycholic acid, lathosterol, (*R*)‐3‐methyl‐2‐oxo‐pentanoic acid, Ala Asn Ile Lys, glycerophospho‐*N*‐palmitoyl ethanolamine, kalkitoxin thioamide alcohol, 11β‐prostaglandin F_2α_‐d_4_, omega‐3 arachidonic acid, and alpha‐pyrrolidinopropiophenone) were significantly up‐regulated and the levels of carnitine and Trp Ala Arg were significantly down‐regulated in males of the high‐dose group.

**Table 9:** Serum differential metabolites of male rats between high‐dose group and blank control group

No.	m/z	Time	Metabolite	VIP	*p*	FC
1	357.2786	6.79	Docosahexaenoic Acid ethyl ester	6.56	0.042	2.44
2	162.1125	0.72	Carnitine	5.07	0.003	0.65
3	391.2854	6.78	Deoxycholic acid	4.06	0.039	2.09
4	359.1902	7.93	Cortisone	3.70	0.009	2.44
5	166.0861	1.72	Phenylalanine	3.64	0.004	1.20
6	391.2864	8.11	Isohyodeoxycholic acid	3.62	0.008	2.14
7	369.3513	20.84	Lathosterol	2.31	0.000	1.25
8	129.0561	4.08	(R)‐3‐methyl‐2‐oxo‐Pentanoic acid	2.13	0.032	1.27
9	432.2375	7.71	Trp Ala Arg	2.04	0.009	0.75
10	443.2579	6.60	Ala Asn Ile Lys	1.67	0.004	4.08
11	454.2927	8.87	Glycerophospho‐N‐Palmitoyl Ethanolamine	1.65	0.029	1.55
12	385.2924	13.36	Kalkitoxinthioamide alcohol	1.32	0.000	1.19
13	341.2663	13.39	11b‐Prostaglandin F2a‐d4	1.27	0.000	1.13
14	305.2474	16.89	omega‐3 Arachidonic Acid	1.14	0.003	1.24
15	204.1384	9.56	Alpha‐Pyrrolidinopropiophenone	1.03	0.003	1.08

#### 3.9.9 Cluster analysis of differential metabolites in male rats

As shown in Tables 8 and 9, there were substantial differences in differential metabolites among low‐dose group, the high‐dose group, and the blank control group.

**Figure 9:**
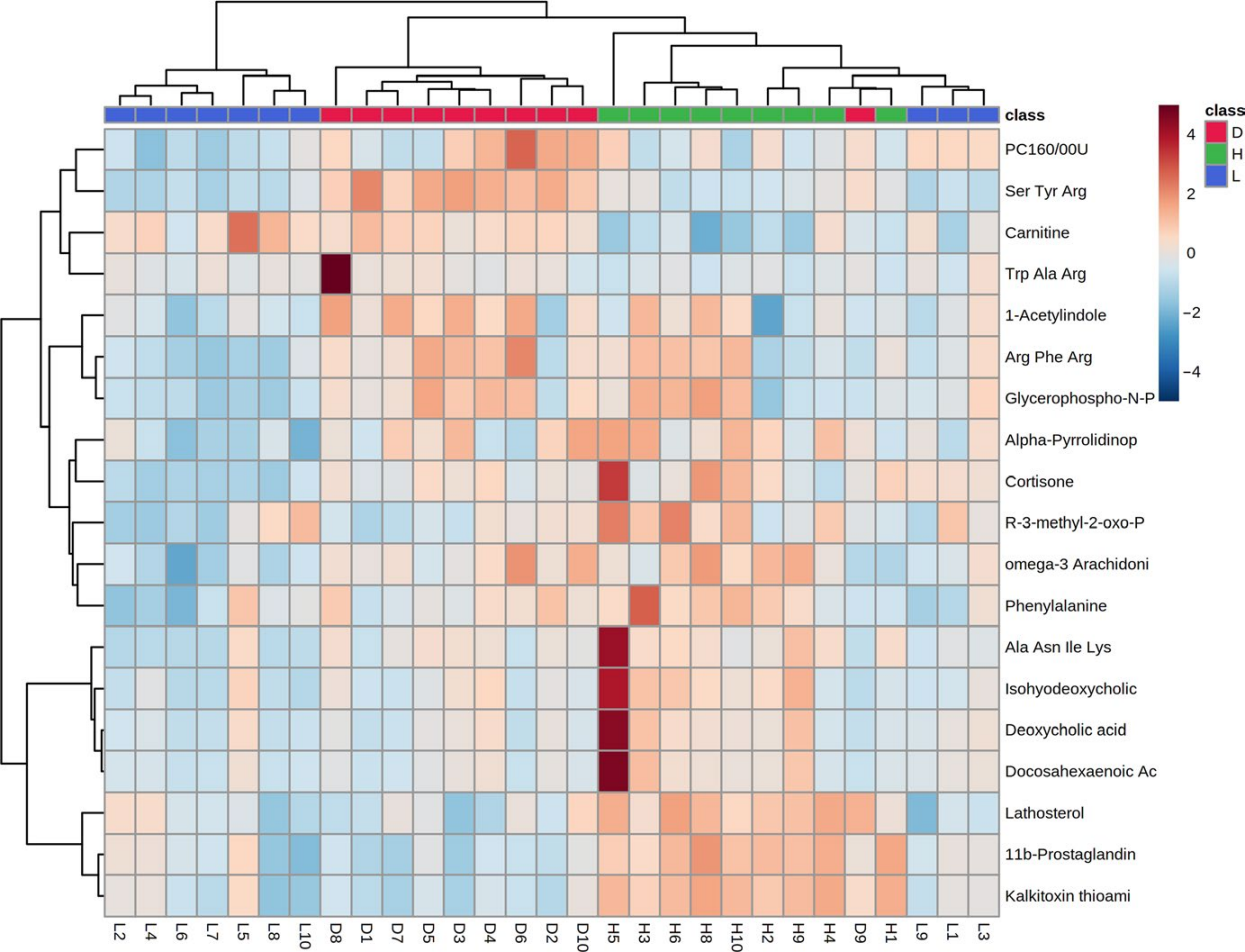
Heat map of serum differential metabolites of male rats in different dose groups

For a more intuitive representation of these differences, a heat map was used to visualize the hierarchical clustering results 19 differential metabolites in the low‐dose group, high‐dose group, and blank control group (where red indicates a higher relative content of the substance and blue indicates a lower relative content). As shown in Figure 9, the blank control group, the high‐dose group, and the low‐dose group showed clear separation with respect to differential metabolites, with division into three main clusters.

#### 3.9.10 Analysis of metabolic pathways in female rats

**Figure 10:**
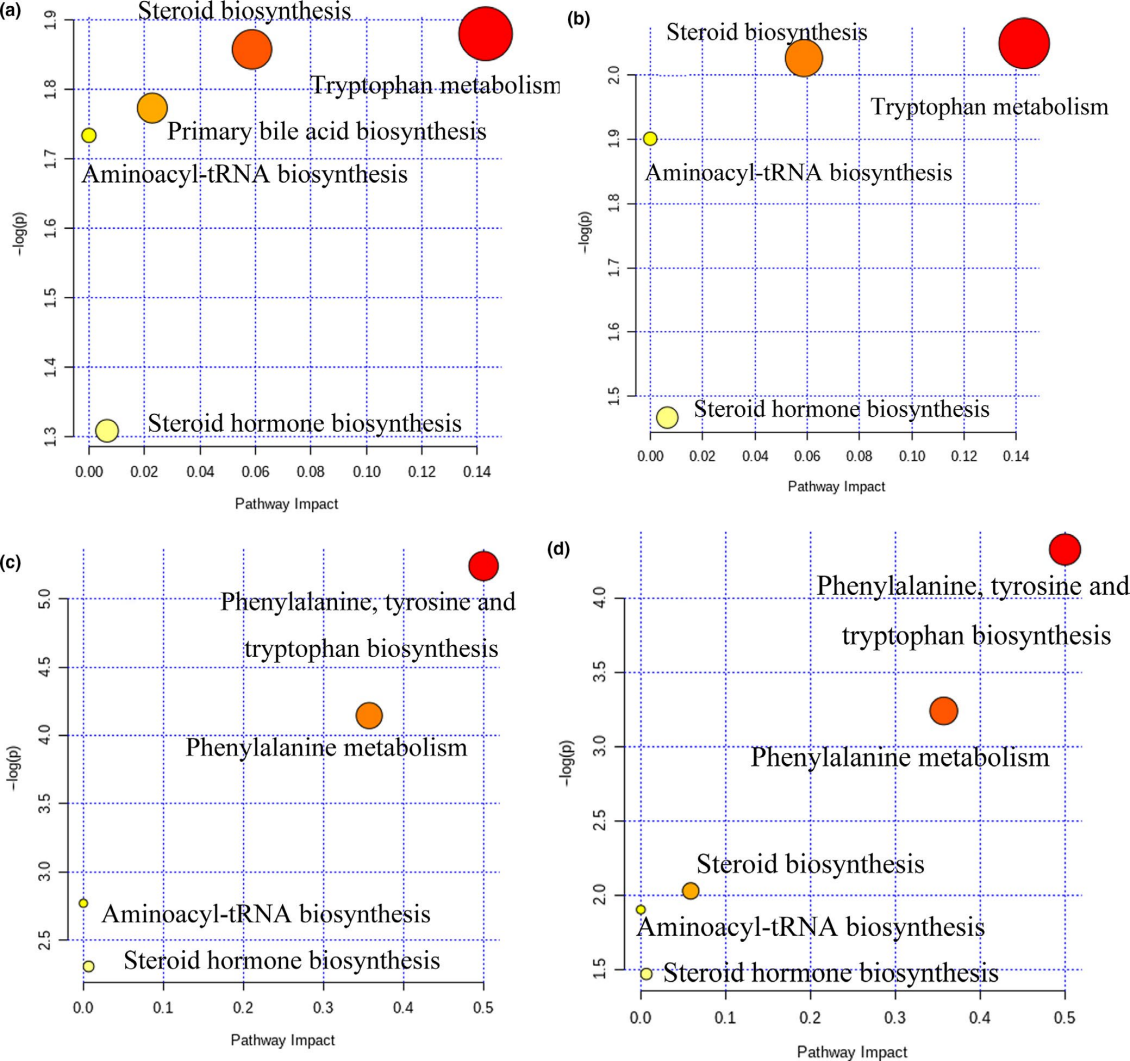
Differential metabolites pathway analysis of female and male rats. a. low‐dose group of female, b. high‐dose group of female, c. low‐dose group of male, and d. high‐dose group of male

The differential metabolites between the low‐dose group and blank control group were imported into the MetaboAnalyst database to analyze the related metabolic pathways. As shown in Figures 10a , 5 metabolic pathways were screened, including tryptophan metabolism, steroid biosynthesis, primary bile acid biosynthesis, aminoacyl‐tRNA biosynthesis, and steroid hormone biosynthesis. However, the ‐log(p) and pathway impact values of the metabolic pathways were small, indicating that the degree of pathway interference is low.

The differential metabolites between the high‐dose group and blank control group were imported into the MetaboAnalyst database to analyze related metabolic pathways. As shown in Figures 10b, 4 metabolic pathways were screened, including tryptophan metabolism, steroid biosynthesis, aminoacyl‐tRNA biosynthesis, and steroid hormone biosynthesis. However, the ‐log (p) and pathway impact values of the above metabolic pathways were small, indicating weak interference.

#### 3.9.11 Analysis of metabolic pathways in male rats

The differential metabolites between the low‐dose group and blank control group were imported into the MetaboAnalyst database to analyze related metabolic pathways. As shown in Figures 10c, 4 metabolic pathways were screened, mainly including phenylalanine, tyrosine, and tryptophan biosynthesis, phenylalanine metabolism, aminoacyl‐tRNA biosynthesis, and steroid hormone biosynthesis. For the aminoacyl‐tRNA biosynthesis and steroid hormone biosynthesis pathways, ‐log(p) > 2 and pathway impact < 0.1 indicated that pathway interference is limited. For the phenylalanine, tyrosine, and tryptophan biosynthesis and phenylalanine metabolism pathways, ‐log(p) > 2 and pathway impact > 0.1 indicate strong pathway interference, suggested that *PLR* effects liver function, mainly via the inhibition of the conversion of phenylalanine to tyrosine and tryptophan or the inhibition of phenylalanine metabolism (Xu et al., 2012; Zheng et al., 2020).

The differential metabolites between the high‐dose group and blank control group were imported into the MetaboAnalyst database to analyze related metabolic pathways. As shown in Figures 10d, 5 metabolic pathways were screened, mainly including phenylalanine, tyrosine and tryptophan biosynthesis, phenylalanine metabolism, steroid biosynthesis aminoacyl‐tRNA biosynthesis, and steroid hormone biosynthesis. For steroid biosynthesis, aminoacyl‐tRNA biosynthesis, and steroid hormone biosynthesis, values of ‐log(p) < 2 and pathway impact < 0.1 indicated that the degree of metabolic pathway interference was low. For phenylalanine, tyrosine and tryptophan biosynthesis and phenylalanine metabolism, values of ‐log(p) > 2 and pathway impact > 0.1 indicated high metabolic pathway interference. These results suggested that *PLR* effects liver function, mainly by inhibiting the conversion of phenylalanine to tyrosine and tryptophan or by inhibiting the metabolism of phenylalanine.

## 4 DISCUSSION

In the 30‐day feeding experiment using adolescent rats, *PLR* did not affect routine blood indexes (including white blood cells, red blood cells, hemoglobin, and platelets), supporting the lack of adverse effects on the red blood cell, white blood cell, and platelet systems. *PLR* had a certain degree of influence on biochemical indicators of liver function, including ALP, ALT, and TP in female rats; however, there were no abnormalities in TBIL, DBIL, TBA, AST, LP (a), ADA, AFU, and ALB. Combined with liver organ coefficients and histopathological analyses, *PLR* did not result in organic lesions in the livers of female rats. *PLR* had no significant effect on biochemical indexes of liver function in male rats and did not result in organic lesions in the liver. These results showed that *PLR* had no significant hepatotoxicity under the present experimental conditions and doses. With respect to renal function, *PLR* had no significant effect on BUN, CRE, and UA in male or female rats; combined with the kidney organ coefficient and histopathological examination, *PLR* had no significant effect on kidney function. *PLR* had a significant effect on the sex hormone estradiol in male rats, without affecting testosterone levels. The histomorphology and organ coefficients of the testis suggested that the intake of *PLR* under the present experimental conditions did not affect the development of male sex organs. The histopathological examination of ovary, uterus, and breast showed that the intake of *PLR* under the present experimental conditions did not significantly promote the development of female sex organs. Furthermore, a serum metabolomics analysis showed that *PLR* mainly causes abnormalities in phenylalanine, tyrosine, and tryptophan biosynthesis or phenylalanine metabolism. It has little influence on the steroid hormone biosynthesis pathway. Therefore, the effects of large doses of *PLR* are specific to the liver.

## 5 CONCLUSION

Overall, *PLR* water extract did not significantly promote the precocity of male and female sexual organs and did not alter liver and kidney function. Therefore, *PLR* water extract is relatively safe for adolescent *SD* rats.

## CONFLICT OF INTEREST

The authors declared no potential conflicts of interest with respect to the research, authorship, and publication of this article.

## ETHICAL APPROVAL

The study on mice was approved by the Ethics Committee of the Experimental Animal Science and Technology Center of Jiangxi University of Traditional Chinese Medicine.

## Supporting information

Fig S1

Fig S2

Fig S3

Fig S4

## Data Availability

All datasets presented in this study are included in the article/supplementary material.
