# Supplementary figures and images for: Safety of high‐dose Puerariae Lobatae Radix in adolescent rats based on metabolomics
Source: Food Sci Nutr. 2020 Dec 1;9(2):794–810. doi: 10.1002/fsn3.2044 (PMC7866568; doi:10.1002/fsn3.2044)

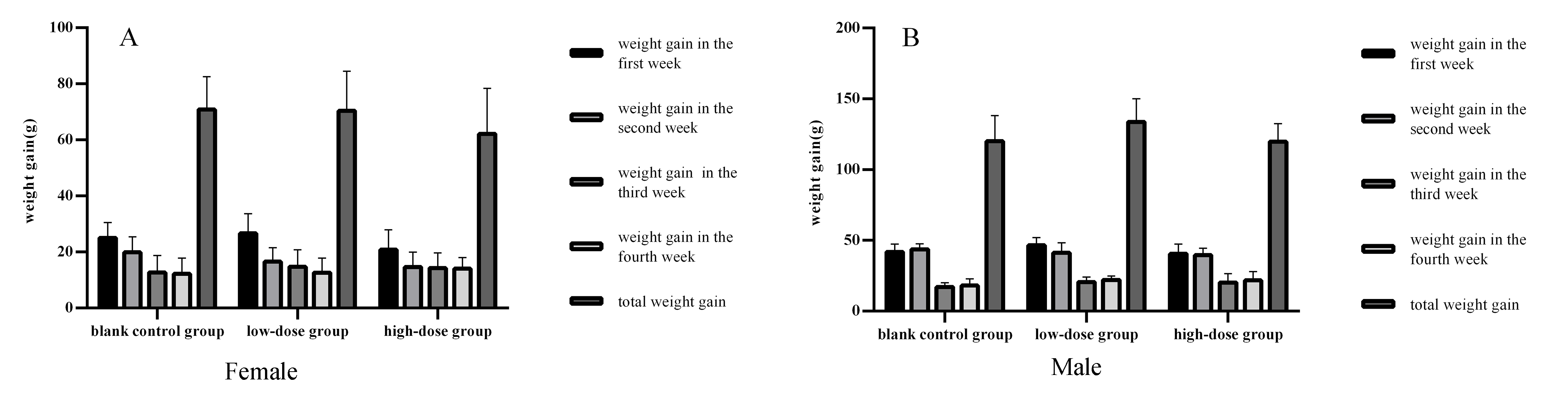

Supplement: Supplementary file 1 — Fig S1 [file FSN3-9-794-s001.tif]

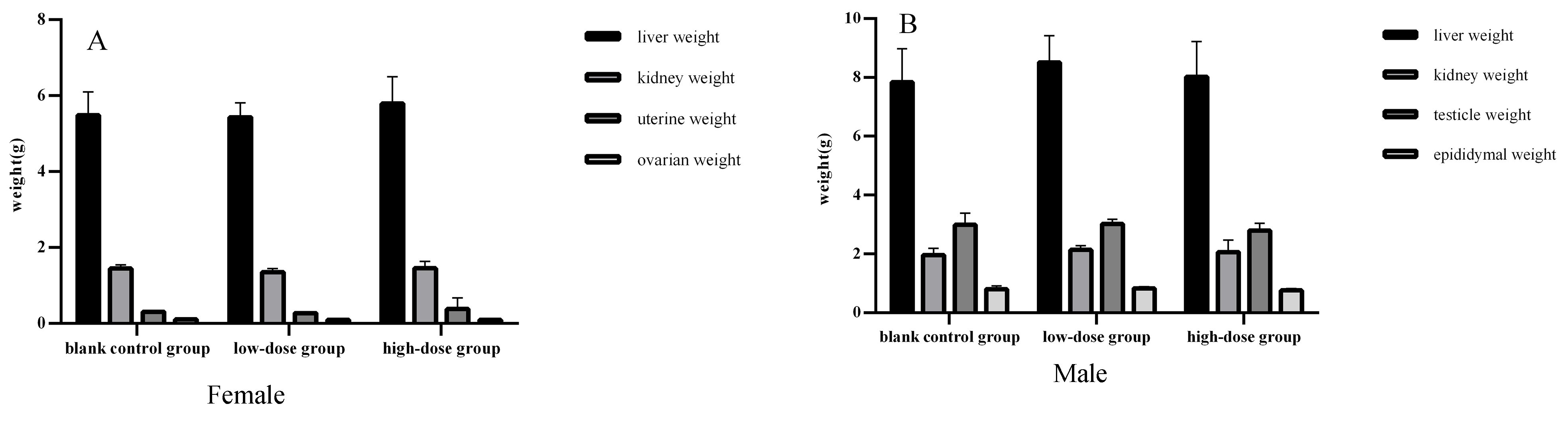

Supplement: Supplementary file 2 — Fig S2 [file FSN3-9-794-s002.tif]

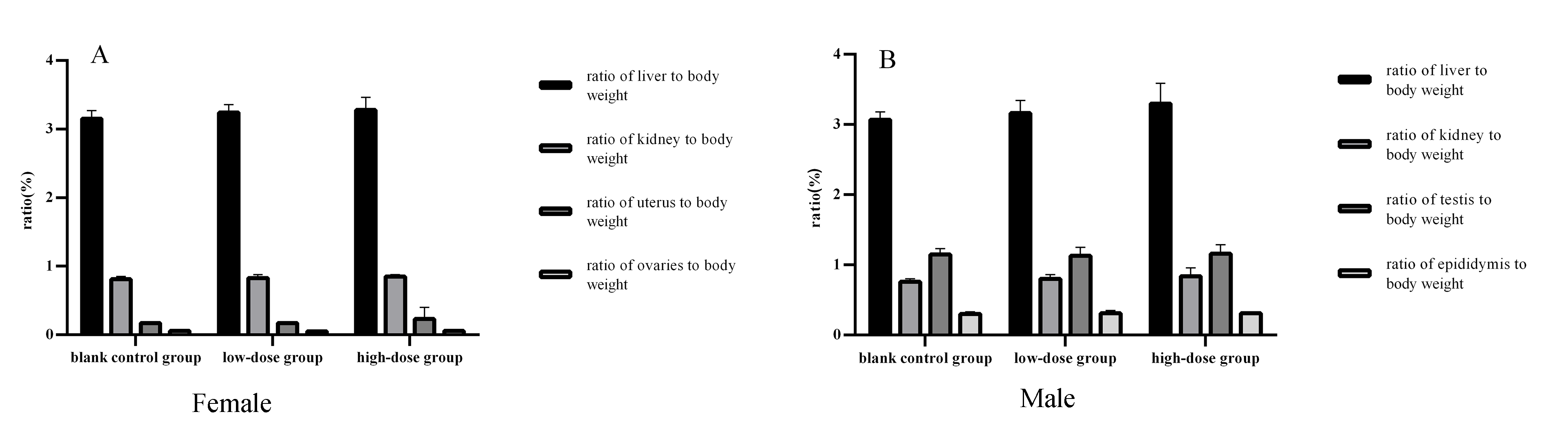

Supplement: Supplementary file 3 — Fig S3 [file FSN3-9-794-s003.tif]
